# Supplementary material for: Stacking Model‐Based Classifiers for Dealing With Multiple Sets of Noisy Labels
Source: Biom J. 2025 Mar 12;67(2):e70042. doi: 10.1002/bimj.70042 (PMC11898607; doi:10.1002/bimj.70042)
Supplement: Supplementary file 1 — Supporting Information [file BIMJ-67-e70042-s001.zip › density-based-ensemble-model-main/competitors_real_data/netlab-master/netlab-master/help/netinit.htm]

Netlab Reference Manual netinit


# netinit

## Purpose

Initialise the weights in a network.

## Synopsis

```
net = netinit(net, prior)
```

## Description

`net = netinit(net, prior)` takes a network data structure
`net` and sets the weights and biases by sampling from a Gaussian
distribution. If `prior` is a scalar, then all of the parameters
(weights and biases) are sampled from a single isotropic Gaussian with
inverse variance equal to `prior`. If `prior` is a data
structure of the kind generated by `mlpprior`, then the parameters
are sampled from multiple Gaussians according to their groupings
(defined by the `index` field) with corresponding variances
(defined by the `alpha` field).

## See Also

`mlpprior`, `netunpak`, `rbfprior`

---

**Pages:**
Index


---

Copyright (c) Ian T Nabney (1996-9)
